# Trends of correlations between serum levels of growth hormone and insulin-like growth factor-I in general practice

**DOI:** 10.3389/fendo.2024.1381083

**Published:** 2024-03-26

**Authors:** Kohei Oguni, Koichiro Yamamoto, Yasuhiro Nakano, Yoshiaki Soejima, Atsuhito Suyama, Ryosuke Takase, Miho Yasuda, Kou Hasegawa, Fumio Otsuka

**Affiliations:** Department of General Medicine, Okayama University Graduate School of Medicine, Dentistry and Pharmaceutical Sciences, Okayama, Japan

**Keywords:** acromegaly, growth hormone (GH), GH deficiency (GHD), insulin-like growth factor (IGF)-I, pituitary gland

## Abstract

Serum levels of growth hormone (GH) and insulin-like growth factor (IGF)-I are crucial in the diagnosis and management of GH-related diseases. However, these levels are affected by nutritional and metabolic status. To elucidate the correlations between GH and IGF-I in various conditions, a retrospective analysis was performed for adult patients in which GH levels were examined by general practitioners during the period from January 2019 to December 2021. Of 642 patients, 33 patients were diagnosed with acromegaly, 21 were diagnosed with GH deficiency (GHD), and 588 were diagnosed with non-GH-related diseases (NGRD). In contrast to the positive correlations found between the levels of GH and IGF-I in patients with acromegaly (*R*=0.50; *P*<0.001) and patients with GHD (*R*=0.39; *P*=0.08), a negative correlation was found in the NGRD group (*R*=-0.23; *P*<0.001). In that group, the results of multivariable analysis showed that GH levels were predominantly influenced by gender and body mass index (BMI), whereas IGF-I levels were modulated by albumin in addition to age and GH. Of note, in the NGRD group, there was an enhanced negative correlation between GH and IGF-I under conditions of BMI < 22 and albumin < 4.0 g/dL (*R*=-0.45; *P*<0.001), and the negative correlation between GH and IGF-I was reinforced by excluding patients with other pituitary diseases and patients taking oral steroids (*R*=-0.51; *P*<0.001 and *R*=-0.59; *P*<0.001, respectively). Collectively, the results indicate that attention should be given to the presence of a negative correlation between serum levels of GH and IGF-I, especially in lean and low-nutritious conditions.

## Introduction

Growth hormone (GH) and insulin-like growth factor (IGF)-I are both essential for growth and metabolism (1). GH deficiency (GHD) is associated with decreases in lean body mass, muscle mass, and bone density, increased fat mass, dyslipidemia, nonalcoholic fatty liver disease (NAFLD), nonalcoholic steatohepatitis (NASH), and decreased quality of life (2, 3). GH is synthesized from somatotrophs in the anterior pituitary gland and its release is mainly induced by GH-releasing hormone (GHRH) and suppressed by somatostatin (1). In patients with acromegaly, it has been shown that there is a linear relationship between GH and IGF-I levels until the GH level reaches 20 ng/mL attaining to plateaus (4). On the other hand, serum IGF-I, secreted by the liver under the influence of GH, inhibits GH secretion directly in somatotrophs and indirectly by stimulating the release of somatostatin (5). Production of IGF-I is mainly regulated by GH, but various factors have an influence on the GH/IGF-I axis. Malnutrition (6), hypothyroidism (7), hepatic insufficiency (8), and poorly controlled diabetes mellitus (9) have been reported to be associated with low serum IGF-I concentrations.

In our previous study, we investigated the relationships between serum IGF-I levels and various laboratory parameters and we found that the level of serum IGF-I had positive correlations with hematopoietic factors but negative correlations with inflammatory and coagulation markers (10). These findings indicated that a decline in serum IGF-I levels with aging could be linked to the increases in inflammatory and procoagulatory factors (10). Considering the increased risk of cardiovascular events with decreased IGF-I with aging (10), the results suggested that serum IGF-I might be a relevant marker for cardiovascular risk.

Measurements of serum levels of GH and IGF-I are important for the diagnosis of GH-related diseases such as acromegaly and GH deficiency (GHD). While GH and IGF-I usually have a parallel relationship in GH-related diseases, elucidation of their interplay in GH-unrelated diseases is crucial for accurately interpreting the GH/IGF-I axis. However, since the interrelationships between levels of serum GH and IGF-I are clinically modified by various nutritious conditions and metabolic factors, careful interpretation is required to evaluate the functional axis composed of GH and IGF-I in clinical practice. The aim of the present study was to clarify the interrelationships between the GH/IGF-I axis and various related physiological parameters in the clinical setting of general practice.

## Methods

### Study subjects and patient groups

Patients in whom GH levels were measured between January 2019 and December 2021 at the Department of General Medicine in Okayama University Hospital were enrolled in this cross-sectional study. Patients who were younger than 18 years of age and patients who were receiving GH replacement therapy were excluded from this study. The first GH level measured during the study period was used for analysis. The decision for measurements of GH and IGF-I was made by individual physicians. Participants were divided into three groups: an acromegaly group, a growth hormone deficiency (GHD) group, and a non-GH-related disease (NGRD) group. Patients in the acromegaly and GHD groups were patients who met the diagnostic criteria for acromegaly and GHD defined by the Japan Endocrine Society, and the other patients were assigned to the NGRD group. The NGRD group was further categorized by excluding patients with pituitary diseases other than acromegaly (*exclusion A*) and patients taking oral steroids including glucocorticoids (11) and estrogens (12) that affect evaluation of the GH/IGF-I axis (*exclusion B*). This study was approved by the Ethical Committee of Okayama University Hospital and adhered to the Declaration of Helsinki (No. 2207-012).

### Analysis of laboratory parameters

Data on age, sex, and body mass index (BMI) were obtained from hospital medical records. Values for the following blood biochemical parameters were also obtained: GH, IGF-I, albumin, total bilirubin, aspartate aminotransferase (AST), alanine aminotransferase (ALT), urea nitrogen, creatinine (Cre), glucose (Glu), hemoglobin A1c (HbA1c), triglycerides (TG), low-density lipoprotein cholesterol (LDL), high-density lipoprotein cholesterol (HDL), C-reactive protein (CRP), thyrotropin (TSH), and cortisol.

The levels of endocrine parameters were determined by using the auto-analyzer system Cobas 8000 (F. Hoffmann-La Roche AG, Basel, Switzerland) at the Central Laboratory of Okayama University Hospital. Serum GH and IGF-I were measured by Elecsys GH and Elecsys IGF-I kits (F. Hoffmann-La Roche AG), respectively, in which the IGF-I standard deviation score (SDS) was calculated on the basis of age- and sex-matched healthy Japanese subjects (13).

### Statistical analysis

Characteristics of the participants are presented using medians and interquartile ranges (IQRs) for continuous variables and frequencies for categorical variables. The Kruskal-Wallis test for continuous variables and Fisher’s exact test for categorical variables were used to analyze subgroup differences. Continuous variables that showed a significant difference in the Kruskal-Wallis test were subjected to the Steel-Dwass test. GH and IGF-I levels were compared using the Mann Whitney U test and Spearman’s correlation for continuous values. In the NGRD group, multiple regression model analysis was performed with GH and IGF-I as dependent variables. Age, sex, BMI, total bilirubin, albumin, Cre, Glu, LDL, CRP, TSH, and cortisol, which are known factors affecting GH and IGF-I, were used as explanatory variables.

Missing continuous covariates included in the regression model were imputed using multiple imputation methods. Multicollinearity was assessed using variance inflation factor, and variables highly correlated with other independent variables were excluded from the final multiple regression model. In the multiple regression model, GH and IGF-I levels were natural log-transformed to provide normality in the regression residuals. Beta coefficients were back-transformed indicating percent increase by one unit increase in corresponding covariates. The NGRD group was divided into four subgroups using BMI and albumin, which were significant explanatory variables in the multivariate regression model, and the correlations between GH and IGF-I were examined. The cutoff for BMI was 22, which is the standard body weight in Japan, and that for albumin was 4.0 g/dL, which is the normal low value. Statistical significance was set at *P*<0.05. Statistical analyses were performed with R statistical software (version 4.3.2).

## Results

### Baseline characteristics of the patients’ groups

Among the 686 patients whose GH levels were measured during the study period, we excluded 26 patients under the age of 18 years and 18 patients who were on GH replacement therapy. The remaining 642 eligible participants were divided into three groups: an acromegaly group (n=33), a GHD group (n=21), and an NGRD group (n=588). Of the 588 patients in the NGRD group, 103 patients were diagnosed with pituitary dysfunctions other than acromegaly and GHD. Ten patients in the NGRD group were treated with oral estrogens and 55 patients in the NGRD group were treated with oral glucocorticoids. All of the patients except one patient in the acromegaly group were in postoperative conditions and/or receiving medical therapy. The baseline characteristics of the patients are shown in **Table 1**. Median values of BMI were 25.1 in the acromegaly group, 24.7 in the GHD group, and 22.4 in the NGRD group, and median BMI was significantly lower in the NGRD group than in the acromegaly group (*P*<0.001). Median albumin levels were 4.2 g/dL in all three groups. The GHD group showed higher levels of AST, ALT, and CRP and lower levels of HDL.

**Table 1:** Basal characteristics of the patients in each group.

	Acromegaly (n=33)	GHD (n=21)	NGRD (n=588)	Defect (%)	*P* values
**Age (years)**	57 [45-68]	57 [44-69]	49 [35-63]	0.0	0.05
**Male sex, n (%)**	15 (46)	14 (67)^†^	221 (38)^†^	0.0	*<0.05^†^
**BMI**	25.1 [23.2-28.1]^†^	24.7 [21.1-26.5]	22.4 [19.8-26.0]^†^	3.1	***<0.001^†^
**GH (ng/mL)**	1.56 [0.70-2.65]^†‡^	0.11 [0.04-0.26]^†§^	0.42 [0.12-1.36]^‡§^	0.0	***<0.001^†‡^, **<0.01^§^
**IGF-I (ng/mL)**	236 [170-281]	56 [31-94]	125 [88-169]	3.4	***<0.001
**IGF-I SDS**	2.30 [0.80-3.10]	-2.90 [-4.30, -2.20]	-0.80 [-1.70-0.10]	3.4	***<0.001
**Albumin (g/dL)**	4.20 [4.07-4.40]	4.15 [3.80-4.40]	4.20 [3.90-4.50]	2.0	0.61
**T-Bil (mg/dL)**	0.75 [0.60-0.84]	0.62 [0.55-0.72]	0.65 [0.49-0.88]	3.3	0.34
**AST (U/L)**	17 [15-22]^†^	26 [20-36]^†‡^	20 [16-25]^‡^	1.6	***<0.001^†^, **<0.01^‡^
**ALT (U/L)**	14 [12-17]^†^	31 [18-38]^†‡^	17 [12-28]^‡^	1.2	**<0.01^†^, *<0.05^‡^
**BUN (mg/dL)**	14.5 [11.6-16.7]	13.4 [11.5-14.2]	12.5 [10.3-15.5]	0.8	0.15
**Cre (mg/dL)**	0.69 [0.62-0.79]	0.77 [0.69-0.96]^†^	0.69 [0.60-0.81]^†^	0.6	*<0.05^†^
**Glucose (mg/dL)**	103 [92-110]	107 [97-123]	100 [92-113]	14.5	0.39
**HbA1c (%)**	6.10 [5.70-6.30]^†^	5.70 [5.47-6.50]	5.60 [5.40-6.10]^†^	24.3	**<0.01^†^
**TG (mg/dL)**	107 [92-148]	126 [103-215]	104 [66-165]	18.2	0.20
**LDL (mg/dL)**	123 [113-143]	129 [104-161]	116 [92-143]	10.3	0.07
**HDL (mg/dL)**	62 [51-87]	43 [36-62]^†^	61 [49-75]^†^	22.9	*<0.05^†^
**CRP (mg/dL)**	0.04 [0.02-0.09]^†^	0.26 [0.08-1.13]^†‡^	0.07 [0.03-0.20]^‡^	4.0	**<0.01^†‡^
**TSH (µIU/mL)**	0.88 [0.60-1.42]^†^	0.75 [0.01-1.89]^‡^	1.54 [1.00-2.51]^†‡^	1.9	***<0.01^†^, **<0.01^‡^
**Cortisol (µg/dL)**	6.70 [5.85-8.80]	7.50 [2.55-14.45]	8.20 [5.80-11.30]	5.0	0.16

Reported counts (proportions) for categorical variables and medians (interquartile ranges) for continuous variables. Values were statistically analyzed by the Kruskal-Wallis test and Steel-Dwass test for continuous variables and by Fisher’s exact test for categorical variables. Significant values were set at *P<0.05, **P<0.01 and ***P<0.001 between the marked groups indicated as cross symbols. GHD, growth hormone deficiency; NGRD, non-growth-hormone-related disease; BMI, body mass index; GH, growth hormone; IGF-I, insulin-like factor-I; SDS, standard deviation score; Alb, albumin; T-Bil, total bilirubin; AST, aspartate aminotransferase; ALT, alanine aminotransferase; BUN, blood urea nitrogen; Cre, creatinine; Glu, glucose; HbA1c, hemoglobin A1c; TG, triglycerides; LDL, low-density lipoprotein cholesterol; HDL, high-density lipoprotein cholesterol; CRP, C-reactive protein; TSH, thyrotropin.

### Correlation between serum levels of GH and IGF-I

**Figure 1:**
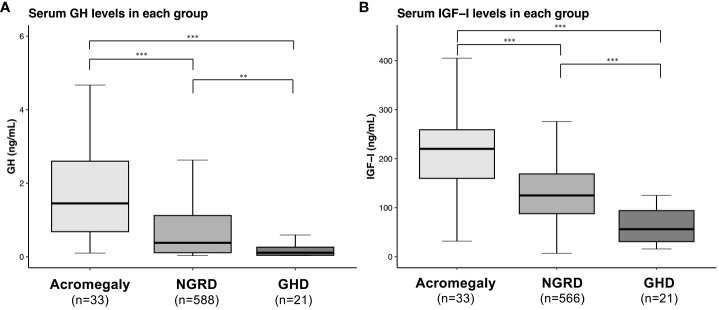
Serum levels of GH and IGF-I in patients with GH-related and GH-unrelated diseases. Comparisons of GH **(A)** and IGF-I **(B)** levels in patients in the acromegaly, non-GH-related disease (NGRD) and growth hormone deficiency (GHD) groups are shown. In each panel, boxplots show the median (horizontal bar in the box), interquartile range (box), and the maximum and minimum values within 1.5 times the interquartile range (horizontal bars outside the box). The Steel-Dwass test was used to illustrate the comparison of GH and IGF-I values among the groups. Significant values were set at **P<0.01 and ***P<0.001.

**Figure 2:**
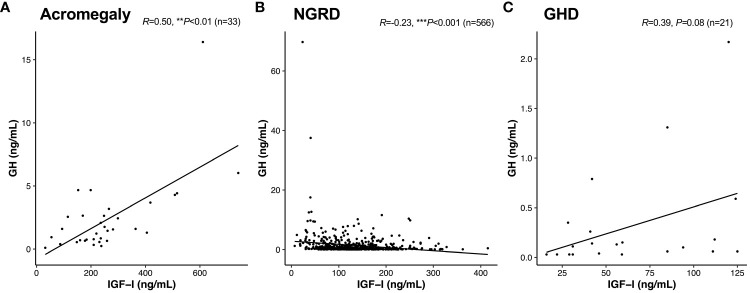
Interrelationships between serum levels of GH and IGF-I in patients with GH-related and GH-unrelated diseases. Correlations between GH and IGF-I in the acromegaly **(A)**, non-GH-related disease [NGRD; **(B)**], and growth hormone deficiency [GHD; **(C)**] groups were analyzed. We regarded **P<0.01 and ***P<0.001 as statistically significant correlations.

As shown in **Figure 1**, serum GH and IGF-I levels were significantly higher in the acromegaly group and significantly lower in the GHD group (*P*<0.001: Acromegaly vs. GHD). Serum GH levels in the NGRD group were significantly different from those in the acromegaly group (*P*<0.001) and GHD group (*P*<0.01) (**Figure 1A**), and serum IGF-I levels were also different in the acromegaly group (*P*<0.001) and GHD group (*P*<0.001) (**Figure 1B**). As shown in **Figure 2**, a positive correlation between serum GH and IGF-I levels was found in the acromegaly group (*R*=0.50; *P*<0.001; **Figure 2A**) and a non-significant but positive correlation was also found in the GHD group (*R*=0.39; *P*=0.08; **Figure 2C**). On the other hand, a mild but significant negative correlation between serum levels of GH and IGF-I was found in the NGRD group (*R*=-0.23; *P*<0.001; **Figure 2B**).

### Regression analyses of serum GH and IGF-I levels in the NGRD group

**Figure 3:**
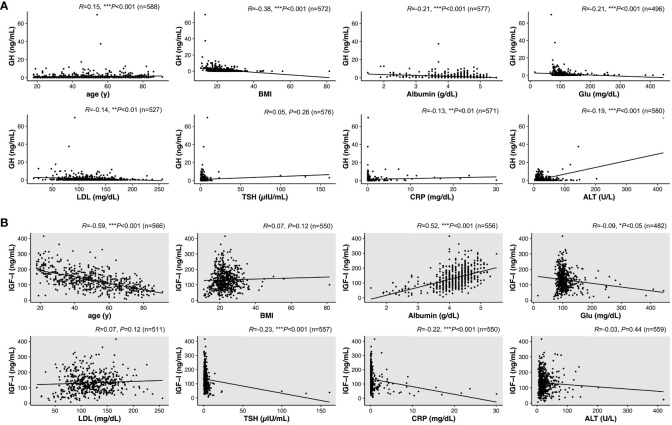
Interrelationships between serum levels of GH or IGF-I and various factors in patients with non-GH-related disease (NGRD). Correlations of serum levels of GH **(A)** and IGF-I **(B)** with various factors including age, body index (BMI), albumin, glucose (Glu), low-density lipoprotein cholesterol (LDL), thyrotropin (TSH), C-reactive protein (CRP), and alanine aminotransferase (ALT) were analyzed in NGRD patients. Significant levels were set at *P<0.05, **P<0.01 and ***P<0.001.

We next investigated correlations of various parameters with serum levels of GH and IGF-I in NGRD patients. As shown in **Figure 3**, by univariate analysis, serum GH levels were found to be positively correlated with age (*R*=0.15; *P*<0.001) and negatively correlated with BMI (*R*=-0.38; *P*<0.001), albumin (*R*=-0.21; *P*<0.001), Glu (*R*=-0.21; *P*<0.001), LDL (*R*=-0.14; *P*=0.002), CRP (*R*=-0.13; *P*=0.001), and ALT (*R*=-0.19; *P*<0.001) (**Figure 3A**). On the other hand, as shown in **Figure 3**, serum IGF-I levels were positively correlated with albumin (*R*=0.52; *P*<0.001) but negatively correlated with age (*R*=-0.59; *P*<0.001), Glu (*R*=-0.09; *P*=0.04), TSH (*R*=-0.23; *P*<0.001), and CRP (*R*=-0.22; *P*<0.001) (**Figure 3B**).

In a multivariable model for the NGRD group (**Table 2**), factors associated with higher GH levels were age (10% relative increase in GH [95% CI; 1%-19%] per 10 year increase, *P*=0.02), total bilirubin (16% relative increase in GH [2%-31%] per 1 mg/dL increase, *P*=0.02), and cortisol (20% increase in GH [6%-36%] per 5 µg/dL increase, *P*<0.01), while factors associated with lower GH levels were male sex (59% decrease in GH [48%-67%], P<0.001), BMI (33% decrease in GH [26%-40%] per 5 increase, *P*<0.001), and LDL (4% decrease in GH [1%-7%] per 10 mg/dL increase, *P*<0.01) (**Table 2A**).

**Table 2:** Multiple regression analysis for factors related to GH and IGF-I.

A) Multiple regression model to predict factors associated with GH in the NGRD group:		
	Adjusted β-coefficient	*P* values
**Age (per 10-year increase)**	**1.10 (1.01-1.19)**	*<0.05
**Male sex**	**0.41 (0.33-0.52)**	***<0.001
**BMI (per 5 increase)**	**0.67 (0.60-0.74)**	***<0.001
IGF-I (per 50 ng/mL increase)	0.89 (0.80-1.00)	0.06
**T-Bil (per 1 mg/dL increase)**	**1.16 (1.02-1.31)**	*<0.05
Cre (per 1 mg/dL increase)	1.17 (0.86-1.60)	0.31
Glu (per 50 mg/dL increase)	0.90 (0.76-1.05)	0.18
**LDL (per 10 mg/dL increase)**	**0.96 (0.93-0.99)**	**<0.01
CRP (per 5 mg/dL increase)	1.20 (0.93-1.56)	0.16
TSH (per 5 µIU/mL increase)	1.04 (0.98-1.10)	0.23
**Cortisol (per 5 µg/dL increase)**	**1.20 (1.06-1.36)**	**<0.01
B) Multiple regression model to predict factors associated with IGF-I in the NGRD group:		
	Adjusted β-coefficient	*P* values
**Age (per 10-year increase)**	**0.88 (0.86-0.90)**	***<0.001
Male sex	1.03 (0.96-1.10)	0.47
**BMI (per 5 increase)**	**1.04 (1.01-1.07)**	**<0.01
**GH (per 1 ng/mL increase)**	**0.98 (0.97-0.99)**	***<0.001
**Alb (per 1 g/dL increase)**	**1.34 (1.24-1.45)**	***<0.001
T-Bil (per 1 mg/dL increase)	0.99 (0.96-1.03)	0.66
**Cre (per 1 mg/dL increase)**	**1.12 (1.02-1.22)**	*<0.05
Glu (per 50 mg/dL increase)	0.97 (0.93-1.01)	0.17
LDL (per 10mg/dL increase)	1.00 (0.99-1.01)	0.79
CRP (per 5 mg/dL increase)	0.93 (0.86-1.01)	0.09
**TSH (per 5 µIU/mL increase)**	**0.98 (0.96-1.00)**	*<0.05

Adjusted β-coefficient values from the multiple regression model are shown as percent changes in GH (A) and IGF-I (B) for a unit change in the covariate. The values in bold indicate statistical significance. Significant values were set at *P<0.05, **P<0.01 and ***P<0.001. BMI, body mass index; GH, growth hormone; IGF-I insulin-like factor-I; Alb, albumin; T-Bil, total bilirubin; Cre, creatinine; Glu, glucose; LDL, low-density lipoprotein cholesterol; CRP, C-reactive protein; TSH, thyrotropin.Bold words mean statistically significant.

As shown in **Table 2**, factors associated with higher IGF-I levels in the NGRD group were BMI (4% increase in IGF-I [1%-7%] per 5 increase, *P*<0.01), albumin (34% increase in IGF-I [24%-45%] per 1 g/dL increase, *P*<0.001), and Cre (12% increase in IGF-I [2%-22%] per 1 mg/dL increase, *P*=0.02), while factors associated with lower IGF-I levels in the NGRD group were age (12% decrease in IGF-I [10%-14%] per 10 year increase, P<0.001), GH (2% decrease in IGF-I [1%-3%] per 1 ng/mL increase, *P*<0.001), and TSH (2% decrease in IGF-I [0%-4%] per 5 µIU/mL increase, *P*=0.01) (**Table 2B**).

**Figure 4:**
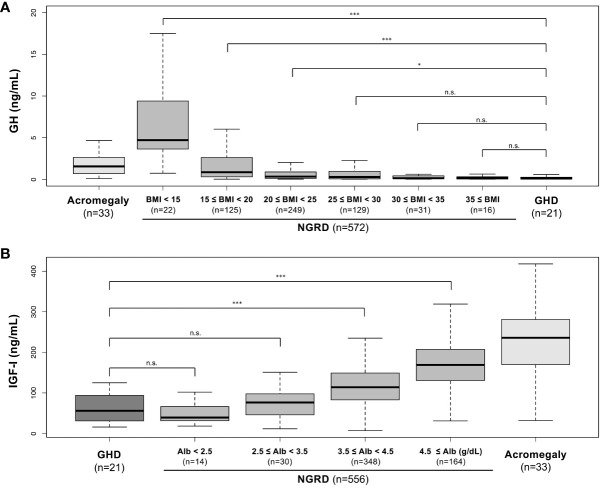
Serum levels of GH and IGF-I in patients with GH-related and non-GH-related diseases (NGRD) stratified by BMI and serum albumin levels. **(A)** Comparison of serum GH levels in each subgroup classified by the levels of BMI in the NGRD group with the acromegaly and GHD groups. The Steel-Dwass test was used to illustrate the comparison of GH values in the GHD group with those in the NGRD group stratified by BMI. **(B)** Comparison of serum IGF-I levels in each subgroup classified by the levels of serum albumin (Alb) in the NGRD group with the acromegaly and GHD groups. The Steel-Dwass test was used to illustrate the comparison of IGF-I values in the GHD group with those in the NGRD group stratified by albumin. An explanation of boxplots of each panel is shown in the legend of **Figure 1**. Significant values were set at *P<0.05 and ***P<0.001. n.s., not significant.

Given the distinct negative correlation between serum GH and BMI levels and a strong positive correlation between serum IGF-I and albumin, the NGRD group was analyzed by stratifying BMI and albumin as shown in **Figure 4**. A comparison of GH levels in the NGRD group with BMI less than 25 and GH levels in the GHD group showed that serum GH levels were significantly lower in the GHD group (*P*<0.05 to *P*<0.001) (**Figure 4A**). Also, serum IGF-I levels in the GHD group were significantly lower than those in the NGRD group with albumin higher than 3.5 g/dL (*P*<0.001) (**Figure 4B**). Subsequent analysis showed no significant difference between serum GH levels in the NGRD group with BMI exceeding 25 and serum GH levels in the GHD group (**Figure 4A**). There was also no significant difference between serum IGF-I levels in the NGRD group with albumin levels below 3.5 g/dL and serum IGF-I levels in the GHD group (**Figure 4B**).

**Figure 5:**
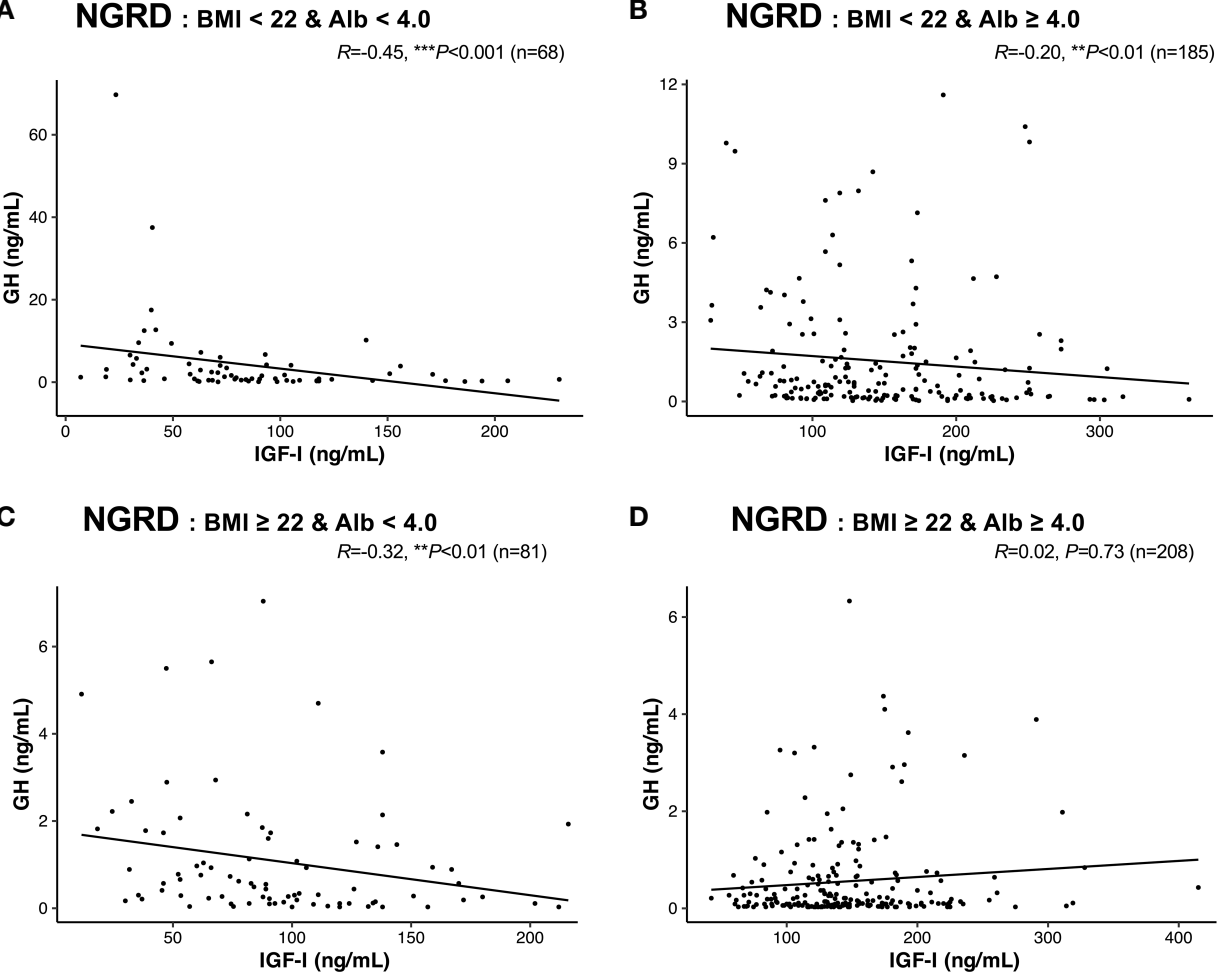
Interrelationships between serum levels of GH and IGF-I in selected patients with non-GH-related diseases (NGRD). Correlations between serum GH and IGF-I levels in the NGRD group divided into 4 subgroups based on the patients’ body mass index (BMI) and serum albumin (Alb) levels are shown: **(A)** BMI < 22 and Alb < 4.0 g/dL, **(B)** BMI < 22 and Alb ≥ 4.0 g/dL, **(C)** BMI ≥ 22 and Alb < 4.0 g/dL, and **(D)** BMI ≥ 22 and Alb ≥ 4.0 g/dL. Significant values were set at **P<0.01 and ***P<0.001.

Finally, we rearranged the interrelationships between serum GH and IGF-I levels in the NGRD subgroup based on standard BMI and serum albumin levels, and we found that in patients with BMI less than 22 and albumin levels below 4.0 g/dL, the significant negative correlation between serum GH and IGF-I levels was enhanced (*R* = -0.45, *P*<0.001; **Figure 5A**). The negative correlations between serum GH and IGF-I levels were clearly impaired in the NGRD subgroups having BMI less than 22 and albumin levels of 4.0 or higher (*R* = -0.20, *P*<0.01; **Figure 5B**) and having BMI of 22.0 or higher and albumin levels less than 4.0 (*R* = -0.32, *P*<0.01; **Figure 5C**), while the correlation was insignificant in the subgroup having BMI of 22.0 or higher and albumin levels of 4.0 or higher (*R* = 0.02, *P*=0.73; **Figure 5D**).

**Figure 6:**
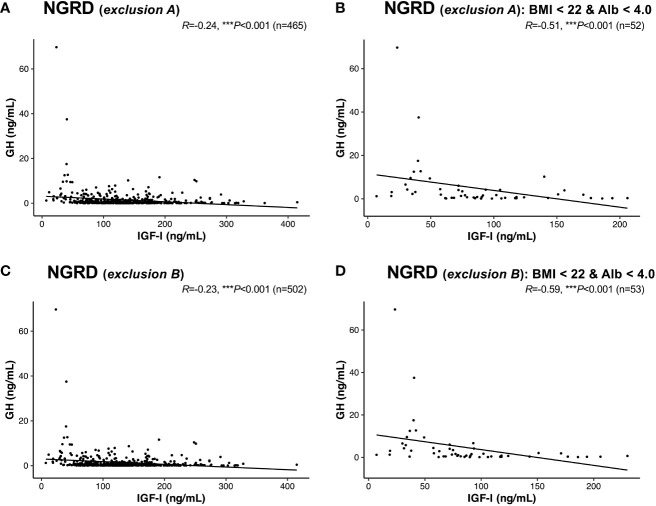
Interrelationships between serum levels of GH and IGF-I in selected patients with non-GH-related diseases (NGRD) excluding patients with other pituitary diseases and patients receiving oral steroids. Correlations between serum GH and IGF-I levels in **(A)** the NGRD group with *exclusion A*, **(B)** an NGRD subgroup (body mass index (BMI) < 22 and serum albumin (Alb) <4.0 g/dL) with *exclusion A*, **(C)** the NGRD group with *exclusion B*, and **(D)** an NGRD subgroup (BMI < 22 and Alb <4.0 g/dL) with *exclusion B. Exclusion A* indicates the exclusion of patients with pituitary diseases other than acromegaly and GH deficiency. *Exclusion B* indicates the exclusion of patients taking oral steroids (glucocorticoids and estrogens). Significant values were set at ***P<0.001.

Furthermore, a negative correlation between serum GH and IGF-I levels was found in the NGRD group in which patients with other pituitary diseases were excluded (*exclusion A*; *R* = -0.24, *P*<0.001; **Figure 6A**), and the correlation was further enhanced in the NGRD subgroup having BMI less than 22 and albumin levels below 4.0 (*R* = -0.51, *P*<0.001; **Figure 6B**). Similarly, a negative correlation between serum GH and IGF-I levels was found in the NGRD group in which patients taking oral steroids were excluded (*exclusion B*: *R* = -0.23, *P*<0.001; **Figure 6C**), and the correlation was further reinforced in the NGRD subgroup having BMI less than 22 and albumin levels below 4.0 (*R*=-0.59; *P*<0.001; **Figure 6D**).

## Discussion

In this cross-sectional study, a negative correlation between GH and IGF-I levels was found in the NGRD group. This correlation was more pronounced in the subgroup of patients with BMI less than 22 and albumin level below 4.0 g/dL. In contrast to the markedly positive correlations found in the acromegaly and GHD groups, a significant negative correlation was found between GH and IGF-I levels in the NGRD group. Moreover, the negative correlations between GH and IGF-I under the same conditions were strengthened in the restricted NGRD groups by excluding patients with other pituitary diseases and those taking oral steroids.

The GH/IGF-I axis is known to be affected by various systemic conditions such as systemic illness, obesity, malnutrition, diabetes mellitus, chronic kidney disease, and liver disease (14). In a state of malnutrition, a condition known as GH resistance is induced. This condition is characterized by suppressed production of IGF-I and increased production of GH, which serve to prevent hypoglycemia (15). GH resistance can also occur in liver disease (16) and systemic illness (17). The presence of GH resistance in the NGRD group could explain the negative correlation found between GH and IGF-I levels in this study. This is further supported by the stronger negative correlation found in the subgroup with lower BMI and albumin levels.

In the multivariable regression analysis, GH levels were found to be associated with age, sex, BMI, total bilirubin, LDL, and cortisol. Of note, sex and BMI seem to have the most significant impact on GH levels. GH is known for its pivotal role in metabolic regulation directly and indirectly via IGF-I synthesized in the liver by GH stimulation (18). Factors that promote GH secretion include sleep, exercise, stress, hypoglycemia, alpha-adrenergic stimulation, beta-adrenergic blockade, and increased blood amino acids, while factors that inhibit GH secretion include IGF-I, hyperglycemia, aging, obesity, alpha-adrenergic blockade, beta-adrenergic stimulation, increased blood free fatty acids, glucocorticoid excess, and thyroid hormone deficiency (5).

The current study further revealed that male patients had 59% lower GH levels than those in female patients and that there was a 33% reduction in GH levels for every 5-unit increase in BMI levels. These results are consistent with results of previous studies showing that male patients and obese patients have lower GH levels (19, 20). A study in which peak GH levels after stimulation with GHRH and arginine were compared in 408 healthy subjects showed a 6.7% decrease in GH levels for each unit increase in BMI and a 5.7% decrease for every 1 year of aging (16). Contrary to previous findings (21, 22), the present study showed that serum GH levels increased by 10% for every 10-year increment in age and by 20% for each 5 µg/dL increase in serum cortisol levels. In the present study, it is possible that factors such as physical and psychologic stress might have affected the cortisol-dependent increase of serum GH in patients in the NGRD group, which is unlikely in healthy individuals.

On the other hand, serum IGF-I levels were revealed to be associated with age, BMI, GH, albumin, Cre and TSH levels in multivariable regression analysis. Serum IGF-I levels were predominantly affected by age, GH, and albumin. IGF-I synthesis is impaired in various conditions including malnutrition, chronic liver failure, serious illness, hypothyroidism, and poorly controlled diabetes (23). In this study, serum IGF-I levels increased by 34% for each 1 g/dL increment in serum albumin and by 4% for every 5-unit rise in BMI and they decreased by 12% for every decade of aging and by 2% for each 1 ng/mL increase in serum GH. The diagnosis of GHD should be made with attention to patients with a low nutritional status, particularly those with serum albumin levels of less than 3.5 g/dL, since such patients had levels of serum IGF-I that were similar to those in the GHD group.

Production of IGF-I in the liver, which is the main source of circulating IGF-I, is promoted by GH binding to the GH receptor (GHR) on hepatocytes (24). For instance, in Laron’s syndrome, which is caused by a genetic mutation in GHR, IGF-I levels are low despite high GH levels due to GHR dysfunction (25). Lowered insulin concentrations during fasting reduce GHR expression and translocation to hepatocytes (26), leading to reduction of IGF-I production by the liver, while administration of GH to fasted rats or humans resulted in a small increase in serum IGF-I concentration (27). In addition to the glucose restriction, it is presumed that protein restriction also reduces IGF-I production by limiting the transcription process following GH binding (28). To maintain normal levels of serum IGF-I, an energy intake of 20 kcal/kg is necessary, along with a protein intake of 0.6 g/kg (29). Hence, it has been recognized that IGF-I can be a useful indicator for the whole nutritional status (30). Taken together with our results showing that the GH/IGF-I correlation was negatively regulated in the NGRD group, changes of systemic IGF-I levels are likely to be the primary factor for the regulation of pituitary secretion of GH in the large population of NGRD patients having a normal GH/IGF-I axis.

There are several limitations of the present study. First, there might be a bias for selecting the patients for measuring serum GH and IGF-I levels, since the decision was made by attending individual physicians. Second, not all individuals in the non-GH-related disease group underwent a hormonal loading test. Consequently, the possibility of GHD could not be entirely excluded. In addition, previous studies have shown that nadir/interpulse GH levels are highly correlated with IGF-I levels in healthy subjects and acromegalic patients (31); however, we used a single measured GH and the timing of blood sampling and dietary condition for endocrine data was not entirely adjusted in each case. Third, even after the two excluded conditions, the patient cohort in this study still included only a small number of individuals with concurrent conditions known to influence the GH/IGF-I axis, such as liver dysfunction, renal impairment, hypothyroidism, adrenocortical hyperfunction, and diabetic conditions. However, the results of the present study would represent the real-world data in a general practice setting that includes patients with a variety of disorders. Finally, the patients with acromegaly in this study were already in a postoperative condition and/or receiving medical therapy, which could influence the GH/IGF-I relationship.

In conclusion, among the patients with non-GH-related diseases, a negative correlation was found between the levels of serum GH and IGF-I, especially in those with low BMI and with low serum albumin levels. This negative correlation is in clear contrast to the expected positive correlation that is typically observed in conditions such as acromegaly and GHD. It is necessary to evaluate the GH/IGF-I correlation by paying attention to the presence of a negative correlation in a wide-ranging population of NGRD patients seen in general practice.

Hence, we need to notice that there are two ways to consider the GH/IGF-I axis, namely the GH-dependent IGF-I production as shown in the GH-related diseases and IGF-I-induced regulation of GH secretion as seen in the NGRD patients. The results of the present study provide an insight for the interpretation of GH and IGF-I levels in various clinical settings and indicate the importance of measuring GH as well as IGF-I even in patients with NGRD.

## Data availability statement

The original contributions presented in the study are included in the article/supplementary material. Further inquiries can be directed to the corresponding author.

## Ethics statement

The studies involving humans were approved by Ethical Committee of Okayama University Hospital (No. 2207-012). The studies were conducted in accordance with the local legislation and institutional requirements. Informed consent from the patients was not necessary due to the anonymization of data. Information regarding the present study was provided on the website of our hospital, and patients who wished to opt out were offered that opportunity.

## Author contributions

KO: Conceptualization, Data curation, Formal analysis, Methodology, Software, Validation, Writing – original draft, Writing – review & editing. KY: Data curation, Formal analysis, Investigation, Methodology, Validation, Writing – original draft, Writing – review & editing. YN: Data curation, Formal analysis, Investigation, Validation, Writing – review & editing, Writing – original draft. YS: Data curation, Investigation, Validation, Writing – review & editing, Writing – original draft. AS: Data curation, Formal analysis, Methodology, Writing – review & editing, Writing – original draft. RT: Data curation, Formal analysis, Writing – review & editing, Writing – original draft. MY: Data curation, Formal analysis, Writing – review & editing, Writing – original draft. KH: Data curation, Formal analysis, Supervision, Writing – review & editing, Writing – original draft. FO: Conceptualization, Supervision, Validation, Writing – original draft, Writing – review & editing, Investigation, Project administration.
